# Functional Capacity and Gut Microbiota Shifts in Heart Failure Patients Following Cardiac Rehabilitation

**DOI:** 10.3390/jcm15103729

**Published:** 2026-05-12

**Authors:** Oliver Boamah Duah, Lufei Young, Claire de La Serre, Haidong Zhu, Hongyan Xu

**Affiliations:** 1 Tanner Health School of Nursing, University of West Georgia, Carrollton, GA 30117, USA; 2 School of Nursing, University of North Carolina, Charlotte, NC 28223, USA; lyoung57@charlotte.edu; 3 College of Veterinary Medicine and Biomedical Sciences, Colorado State University, Fort Collins, CO 80523, USA; claire.de_la_serre@colostate.edu; 4 Georgia Prevention Institute, Augusta University, Augusta, GA 30912, USA; hzhu@augusta.edu; 5 Department of Biostatistics, Data Science and Epidemiology, School of Public Health, Augusta University, Augusta, GA 30912, USA; hxu@augusta.edu

**Keywords:** gut microbiota, heart failure, microbiome, functional capacity, cardiac rehabilitation, microbiome

## Abstract

**Background/Objectives**: Increased functional capacity is associated with healthy gut microbiota composition and improved heart failure (HF) prognosis. Although cardiac rehabilitation (CR) improves functional capacity in HF patients, the association between CR and gut microbiota in HF patients is not well-studied. We explored the relationships between functional capacity and changes in gut microbiota composition in 41 patients with HF participating in CR, using data from a previous repeated measures clinical trial. **Methods**: Functional capacity was evaluated using the six-minute walk distance (6MWD), and the gut microbiota composition was analyzed from fecal samples using 16S rRNA sequencing before CR and again after three months of participation. **Results**: Higher baseline functional capacity (6MWD ≥ 360 m) corresponded with significant shifts in Bacteroidetes abundance after CR. A clinically meaningful improvement in functional capacity (change in 6MWD ≥ 95 m) was associated with increased α-diversity (*p* = 0.04, statistic = 6.42), increased abundance of *Lachnospiraceae UCG 004*, *Lawsonella*, and *Ruminococcus gnavus* group, and a lower abundance of *Microbacterium*. **Conclusions**: These data suggest that cardiac rehabilitation may be associated with differences in gut microbiota composition in HF patients, alongside its association with functional capacity. Additional research is needed to better understand how gut microbial patterns relate to functional capacity in individuals with HF participating in CR.

## 1. Introduction

Cardiac rehabilitation (CR) is initiated to improve HF prognosis after acute hospitalization. It includes behavioral and nutritional changes, physical activity, and medical management to achieve optimal physical, psychological, and social quality of life (QoL) through improved functional capacity [1,2]. HF patients who participate in CR improve their exercise tolerance, daily function, and overall quality of life [3,4]. In cardiac rehabilitation populations, the strong positive association between myocardial strain parameters and functional capacity measures, such as the 6 min walk test and peak oxygen uptake, despite unchanged left ventricular ejection fraction (LVEF), underscores that functional and biological recovery can occur independently of traditional systolic metrics [5].

Emerging evidence indicates that gut microbiota may be a novel biological marker for heart failure pathogenesis and prognosis. Changes in gut microbiota composition observed in individuals with HF, including reduced microbial diversity and increases in pathogenic taxa, have been linked to decreased short-chain fatty acids (SCFAs), higher Trimethylamine N-oxide (TMAO) levels, and elevated inflammatory cytokines [6,7,8,9,10]. Moreso, decreased gut microbiota diversity has been associated with frequent rehospitalizations and poorer LVEF and quality of life [11].

Although the gut microbiota of individuals with heart failure has been described in prior studies, the potential relationship between participation in CR and the subsequent shifts in gut microbiota remains unexplored in this population. Given the association of gut microbiota in HF pathogenesis and evidence suggesting that CR improves functional capacity, we explored the composition of gut microbiota profiles in HF patients before and after participation in CR. We examined the associations between changes in functional capacity and gut microbiota composition before CR and after three months of participation in CR.

## 2. Materials and Methods

### 2.1. Study Population

This study was an exploratory secondary data analysis of a single cohort without a control group. Participants were individuals with HF who had recently been discharged from the hospital and subsequently enrolled in phase II CR. The original study took place at a rural critical access hospital (CAH) between September 2013 and October 2015 and was conducted in accordance with the Declaration of Helsinki. The initial study was approved by the University of Nebraska Medical Center Institutional Review Board and the hospital’s ethics committee. Critical access hospitals are federally designated facilities that operate with fewer than 25 acute care beds and are situated at least 35 miles from the nearest hospital [12].

Participants were eligible for the original study if they were at least 21 years old and had a confirmed diagnosis of heart failure, classified as NYHA class II–IV, or class I with symptoms and at least one heart-failure-related hospitalization or emergency department visit within the prior year. HF patients scheduled for upcoming procedures or surgeries, those with documented liver cirrhosis or renal impairment (serum creatinine >2.0 mg/dL), or those exhibiting depressive symptoms were excluded. For this secondary study, participants were excluded if microbiota data were missing at baseline, after completion of cardiac rehabilitation, or at both time points. Participants exposed to antibiotic therapy three months before the start date or during the period of the study were also excluded from this analysis [13]. A cohort of 41 HF patients who completed the full three-month cardiac rehabilitation program and supplied fecal specimens at both the initial assessment and the three-month follow-up constituted the sample for this secondary analysis.

### 2.2. Power and Sample Size Estimation

Power was estimated with a one-sample *t*-test using the change in Shannon diversity index before and after CR. The study achieved 0.90 power for testing the mean group difference in α-diversity with 41 subjects (mean = 0.30, SD = 0.58). We used the correlation estimates between the change in 6MWT distance and the change in gut microbiota features (*Akkermansia* genus) to estimate that the power for intergroup comparisons would have at least a moderate effect size, the hypothesis Ho: r = 0 against HA: r = 0.42, when α = 0.05.

### 2.3. Exercise Training Protocol

Exercise protocol included individualized aerobic and resistance training. Participants completed supervised aerobic training 3–5 days/week. Session duration ranged from 20 to 60 min and was tailored to each participant’s exercise tolerance. Exercise intensity was guided by heart rate reserve (HRR) and by participants’ rating of perceived exertion (RPE). Moderate intensity corresponded to 40–59% HRR (RPE 12–13) and vigorous intensity to 60-89% HRR (RPE 14–17). Modalities included treadmill walking, cycling, and other continuous or interval-based aerobic exercises such as speed walking. Resistance training was performed 2–3 nonconsecutive days/week at 40–60% of one repetition maximum for 10–15 repetitions across major muscle groups. Frailty and fall risk were assessed using validated functional tests, including the Timed Up and Go, 30 Second Chair Stand, 4 Stage Balance Test, and Berg Balance Scale.

### 2.4. Functional Capacity Assessment

Functional capacity was evaluated before CR and again after three months of CR, using the six-minute walk test (6MWT). For each assessment, participants walked at a self-selected pace for six minutes along a marked 30 m indoor corridor under the supervision of trained medical staff. Total distance covered was recorded in meters. Participants were asked to continue their prescribed medications and to wear comfortable clothing and footwear for the assessment. A member of the cardiac rehabilitation team measured blood pressure, oxygen saturation using pulse oximetry, and heart rate immediately before and after the test.

The 6 min walk test (6MWT) is a validated submaximal exercise assessment widely used to assess functional capacity in patients with heart failure. It serves as a strong prognostic indicator for major clinical outcomes, including hospitalization and mortality, independent of natriuretic peptide levels and New York Heart Association (NYHA) functional status [14,15,16]. Although peak oxygen consumption (VO_2_peak) obtained from cardiopulmonary exercise testing (CPET) is considered the criterion standard for assessing maximal cardiorespiratory fitness, CPET is resource-intensive and may be difficult for HF patients with exercise intolerance to complete [17,18,19]. In contrast, the 6MWT provides a practical, low-cost, and accessible measure of submaximal functional performance that better captures patients’ functional ability in routine daily activities [20,21].

The 6MWT has demonstrated strong reliability and validity in HF populations, including significant correlations with maximal power output and VO_2_-based indices of cardiorespiratory fitness [22]. Furthermore, classification of cardiorespiratory fitness using 6MWT distance is valid for identifying individuals with low fitness, a characteristic common among HF patients [23]. Given its prognostic value, feasibility, and alignment with real-world functional capacity, the 6MWT was used in this study to assess changes in functional capacity before and after cardiac rehabilitation.

#### 2.4.1. Gut Microbiota Analyses

Fecal samples were obtained at the CR center during the original study at both the baseline and three-month time points using self-administered rectal swabs [24]. A research team member showed participants how to collect rectal samples using a visual aid. Participants were instructed to insert the swab roughly an inch into the anal canal and rotate it gently for several seconds. After collection, one swab was placed into a 2 mL cryovial without preservative, while the second was transferred into a matching vial containing Invitrogen RNAlater stabilization solution (Thermo Fisher Scientific, Waltham, MA, USA). All specimens were kept upright in a −80 °C freezer until DNA extraction, after which they were shipped to Novogene Corporation (Sacramento, CA, USA) for 16S rRNA sequencing and operational taxonomic unit analysis.

Bacterial DNA was extracted from fecal swabs using the CTAB/SDS lysis method. All samples were processed using the same extraction kit lot, PCR reagents, and sequencing chemistry, with samples randomized across extraction and PCR batches. DNA integrity and quality were first checked on a 1% agarose gel, after which samples were diluted to 1 ng/μL. The V3–V4 region of the 16S rRNA gene was amplified using the 341F (CCTAYGGGRBGCASCAG) and 806R (GGACTACNNGGGTATCTAAT) primer pair (Novogene Corporation, Sacramento, CA, USA). Amplification was performed with Phusion^®^ High-Fidelity PCR Master Mix (Thermo Fisher Scientific, Waltham, MA, USA) under standard cycling conditions. Resulting amplicons were combined in equal mass proportions and subsequently purified to remove residual primers and nonspecific fragments.

Sequencing libraries were generated using NEBNex t^®^ Ultra DNA Library Pre-Kit for Illumina (San Diego, CA, USA), with quality assessed on a Qubit@ 2.0 Fluorometer (Thermo Fisher Scientific, Waltham, MA, USA) and Agilent Bioanalyzer 2100 system (Agilent Technologies, Santa Clara, CA, USA). Libraries were sequenced on the Illumina platform, generating 250 bp paired-end reads. Reads were assigned to samples, truncated, and merged using FLASH version 1.2.7. Quality filtering of raw tags was performed according to QIIME (V1.7.0) quality control processes to remove low-quality reads, ambiguous bases, and sequences below quality thresholds. Chimeric sequences were identified and removed using UCHIME against the Gold reference database, yielding high-quality effective tags for downstream analysis. Uparse software version 7.0.1001 was used to analyze sequences, assigning those with ≥97% similarity to the same OTUs. Representative sequences for each OTU were screened and annotated using the Greengenes Database based on RDP classifier version 2.2 [25,26].

#### 2.4.2. Downstream Analysis

Gut microbiota analyses were performed using MicrobiomeAnalyst [27,28]. A low count filter was applied at 20% for sequencing errors, and a low variance filter (interquartile range) removed 10% of features. All samples were rarefied to the minimum library size to eliminate variance in sequencing depth. Rarefied OTUs were transformed using relative log expression (RLE) before downstream analysis.

Alpha diversity metrics (observed OTUs, Shannon, Simpson, Fisher, ACE, and Chao1) were calculated. Normalized OTU tables were used for β-diversity analysis. Bray–Curtis’s dissimilarity index, Jensen–Shannon, and Jaccard were calculated at phylum, genus, and species taxonomic levels. Differences between samples at baseline and three months were estimated using Permutational Multivariate Analysis of Variance (PERMANOVA), Permutational Multivariate Analysis of Dispersion (PERDISP), and Analysis of Similarities (ANOSIM). Principal coordinates analysis (PCoA) was applied to β-diversity indices, and LEfSe was performed to clarify dominant bacterial species. Further downstream statistical analysis was performed in R version 4.5.2 using the tidyverse package [29,30]. Categorical parameters were compared by Pearson’s Chi-squared test and numerical parameters by Spearman’s rank correlation, adjusting *p*-values with the Benjamini–Hochberg method to control false discovery rate (FDR). A stepwise regression model and ANOVA analysis were then used to determine which patient characteristics were significantly associated with the observed changes in the relative abundance of gut microbiota features. Kruskal–Wallis’s test was employed to assess the differences in the relative abundance of genera between participants who had clinically significant improvement in their 6MWD and those who did not. Statistical significance was set at α = 0.05 for all comparative analyses.

## 3. Results

### 3.1. Sample Characteristics and Clinical Parameters

Data from 41 HF patients participating in CR were included. Participants were predominantly male (63.4%) and Caucasian (75.6%), with a mean age of 68.0 (SD = 10.2) years. Most were NYHA class II (56.1%). Mean HDL and LDL at baseline were 47.8 (±15.1 mg/dL) and 88.5 (±36.5 mg/dL), respectively. Paired *t*-tests showed no significant differences in BMI, HDL, and Triglycerides before and after CR, nor in serum inflammatory biomarkers, medication regimen, and medical history. However, serum cholesterol (*p* = 0.001) and low-density lipoprotein (*p* = 0.006) decreased after CR. Demographic and clinical variables are summarized in Table 1.

#### 3.1.1. Participant Characteristics and Gut Microbiota Composition

We observed no significant differences in α and β-diversity based on participant characteristics (age, gender, smoking status, comorbidities, and medication use) at baseline. There was a significant decrease in serum cholesterol and triglyceride levels; however, these changes did not correspond to significant changes in either α- or β-diversity.

There were no significant relationships between the changes in relative abundances of Firmicutes, Proteobacteria, and Actinobacteria with age, sex, BMI, or baseline functional capacity (pre-6MWD). However, baseline Firmicutes abundance, baseline functional capacity (r = 0.44, *p* = 0.01), and BMI (r = −0.35, *p* = 0.03) correlated with an increase in Bacteroidetes after CR. Baseline walking distance above 360 m (β = 17,734, *p* = 0.003) and initial Firmicutes abundance (β = 0.56, *p* = 0.027) emerged as independent predictors of the higher post-rehabilitation Bacteroidetes abundance in the stepwise regression model, which accounted for 46% of the variance after adjusting for BMI change. Follow-up ANOVA indicated that both starting Firmicutes levels (*p* = 0.009) and the baseline 6MWD category (*p* < 0.001) were significantly related to differences in Bacteroidetes following completion of CR.

#### 3.1.2. Observed Shifts in Functional Capacity and Gut Microbial Profiles After CR

On average, HF patients showed significant improvement in their physical functional capacity after CR (t = 7.12, df = 28, *p* ≤ 0.001), with a mean increase of 81.51 m in the distance covered on the 6MWT at three months (95% CI = 58–105 m).

We compared OTU abundance at baseline and after three months of CR. While there was a decrease in the abundance of Firmicutes (*p* = 0.001), Proteobacteria increased after three months of CR (*p* = 0.004) in the overall sample after CR. The abundance of Bacteroidetes decreased (*p* = 0.057), and Actinobacteria increased (*p* = 0.11) but not significantly, as shown in Table 2. Likewise, the Shannon and Simpson analysis proved a significant decrease in gut microbiota α-diversity after CR at the genus and species taxonomic levels (*p* = 0.019 and *p* = 0.003, respectively) in the overall sample of 41 participants. For intergroup comparisons, we divided participants into two groups according to whether they had increased their 6MWD by ≥95 m (significant group) or less than 95 m (non-significant group).

**Figure 1:**
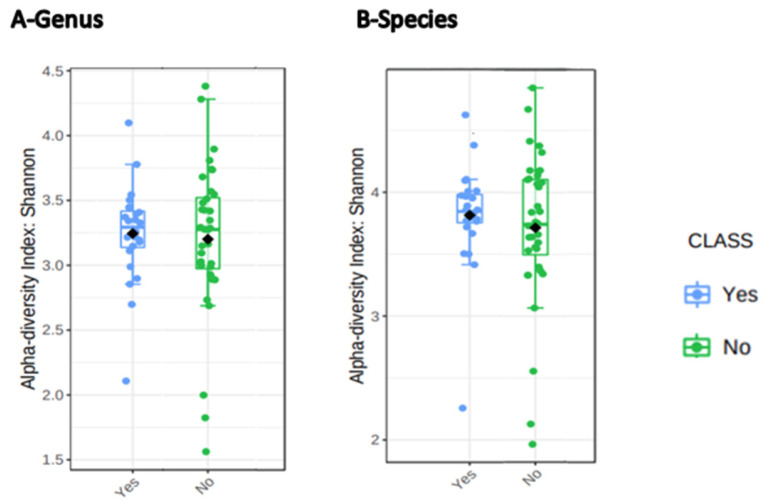
Boxplot depicting alpha diversity stratified by functional capacity change following CR. Note. Participants were grouped into two classes (Yes and No categories). Yes = participants who had a clinically significant change in functional capacity after CR. No = participants with no clinically significant change in functional capacity after CR. ◆ = Mean alpha diversity for each group.

We found that the α-diversity of participants who significantly improved their functional capacity was significantly different and slightly higher than that of those who did not (*p* = 0.04, statistic = 6.42) based on the Kruskal–Wallis test at the genus level (Figure 1). We also observed a higher abundance of Firmicutes and lower Bacteroidetes abundance after CR in participants who significantly improved their functional capacity compared to their counterparts. A Welch two-sample *t*-test was conducted to examine whether the F/B ratio differed between HF patients with significant improvement versus those who had no significant improvement in functional capacity after CR. The results indicated that the difference between groups was not statistically significant, t (24.51) = −1.27, *p* = 0.22, although HF patients with significant improvement showed a higher mean in FB ratio value (M = 1.53) compared to (M = 1.24).

**Figure 2:**
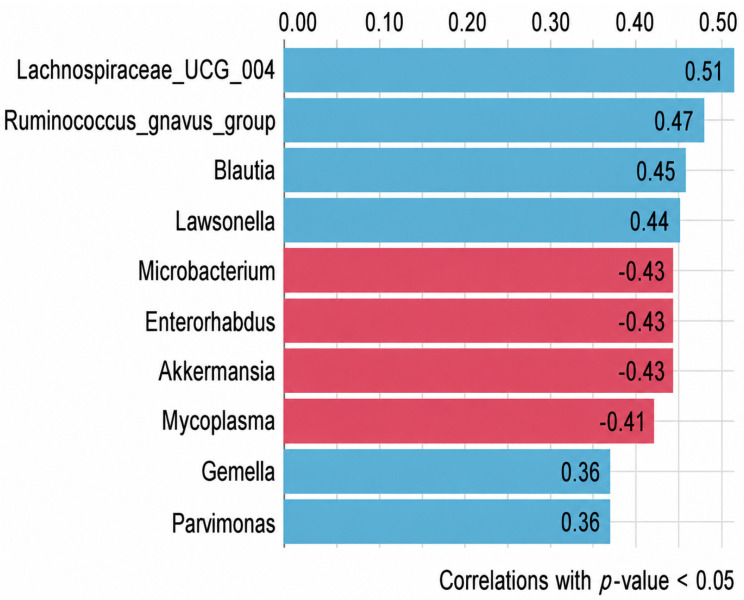
Bar plot showing spearman correlations between change in functional capacity and change in the relative abundance of specific genera after CR. Note. Figures in each bar plot are the correlation coefficient between the specific genera and the change in functional capacity.

Furthermore, we performed a Spearman’s correlation analysis between the change in functional capacity and the change in relative abundance. Changes in functional capacity did not significantly correlate with changes in gut microbiota composition at the phylum level. At the genus level, an increase in functional capacity (Δ6MWD) correlated with an increase in the relative abundance of *Lachnospiraceae_UCG_004*, *Ruminococcus gnavus* group, *Blautia*, *Lawsonella*, *Gemella*, and *Parvimonas*, and decreasing relative abundance of *Microbacterium*, *Enterorhabdus*, *Akkermansia*, and *Mycoplasma* after adjusting *p*-values with the Benjamini–Hochberg method (Figure 2). After adjustment for change in BMI, only *Akkermansia* remained significantly associated with change in functional capacity (*p* = 0.03, r = −43).

**Figure 3:**
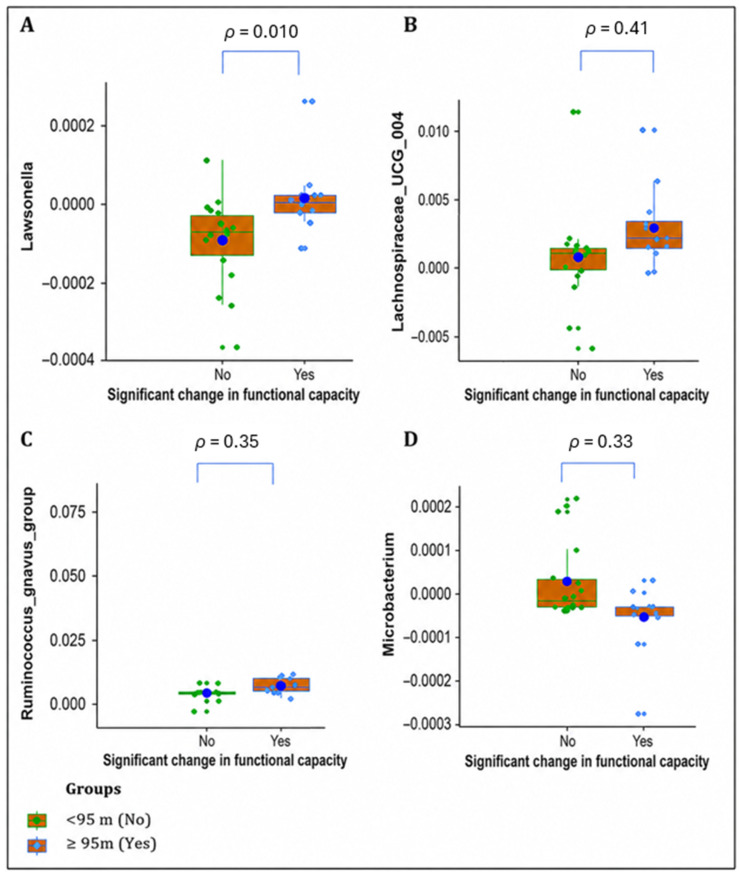
Box plot showing differences in genera associated with functional capacity improvement after CR. (**A**) *Lawsonella*, (**B**) *Lachnospiraceae_UCG_004*, (**C**) *Ruminococcus_gnavus*_group, (**D**) *Microbacterium*; ● = Mean OTU abundance for each group.

As shown in Figure 3, pairwise comparison between groups showed that participants with a significant increase in functional capacity (6MWD ≥ 95 m) had a greater relative abundance of *Lawsonella* (U = 38, *p*_adj_ = 0.010, r = 0.65), *Lachnospiraceae_UCG_004* (U = 53, *p*_adj_ = 0.041, r = 0.51), and *Ruminococcus_gnavus*_group (U = 48, *p*_adj_ = 0.035, r = 0.56). On the other hand, participants with a significant change in functional capacity (6MWD) had a lower relative abundance of *Microbacterium* (U = 167, *p*_adj_ = 0.033, r = 0.55) than those who did not.

## 4. Discussion

In this study, we sought to assess the relationships between functional capacity measured by the 6MWD and gut microbiota composition in patients with HF who participated in CR. We analyzed gut microbiota from fecal samples using 16S rRNA sequencing before and after three months of CR. The baseline gut microbiota profile in our sample was consistent with that in previous studies in which researchers compared gut microbiota composition in HF patients with healthy controls [9,31,32].

Our results demonstrated a robust improvement in functional capacity following cardiac rehabilitation. The mean increase in 6 min walk distance (6MWD) was 81.51 m (*p* < 0.001), exceeding the ~30–70 m range commonly cited as the threshold for clinically meaningful group-level change in HF populations [33,34]. However, we observed a decreased α-diversity after CR participation. The significant reduction in α-diversity from baseline to three months after CR diverged from our initial expectations and from prior work linking higher physical functioning and regular exercise with greater microbial diversity in athletes and healthy older adults [35,36,37]. This contrast highlights individual variability in gut microbiota responses, consistent with cross-sectional evidence showing lower α-diversity in those with more severe heart failure [38]. A careful examination of participant-level patterns showed that the overall decline may have been influenced by four individuals who exhibited pronounced decreases in α-diversity. These participants also showed patterns such as weight gain, reduced functional capacity, or minimal change in functional performance over the study period, with some experiencing more than one of these features. These observations suggest that variability in α-diversity trajectories may reflect broader differences in participants’ clinical or functional responses during the rehabilitation timeframe.

To determine whether individual participants achieved a clinically meaningful improvement, we applied a 95 m criterion based on the work of Hanson and colleagues, who reported that an individual must improve their 6MWD by at least 95 m to surpass test–retest variability with 95% confidence [39]. Using this threshold, between-group comparisons showed that participants who achieved a significant functional gain (Δ6MWD ≥ 95 m) over the three-month CR program exhibited higher α-diversity than those who did not meet this benchmark (*p* = 0.04, statistic = 6.42).

Though we saw an increase in the F/B ratio for participants who demonstrated higher functional capacity, it did not reach the level of significance. These results contrast with those found by Motiani et al., who noted a decrease in the F/B ratio after two weeks of moderate-intensity training in sedentary middle-aged insulin-resistant subjects [40]. Motiani and colleagues found that the reduction in the F/B ratio was driven by a marked rise in Bacteroidetes while Firmicutes remained unchanged, whereas our data showed a higher abundance in Firmicutes in those who significantly improved their physical function. Research involving patients with HF has demonstrated a significantly lower F/B ratio compared to healthy controls [31,41]. Although this association is not found in all studies, Mayerhofer et al. found a link between a lower F/B ratio and poorer HF prognosis and clinical outcomes [31,42].

Also, *Ruminococcus gnavus* and *Lawsonella* abundance were higher in HF patients who had a significant increase in functional capacity. While some *Ruminococcus* species are associated with butyrate production, a recent study linked *Ruminococcus gnavus* with glucorhamnan polysaccharide that stimulates dendritic cells to release tumor necrosis factor-α [43]. Likewise, *Lawsonella* species are naturally occurring in the upper respiratory tract and skin but have been associated with opportunistic vascular graft infections and abscess [44,45]. However, these findings in the literature lack functional validation, and as such, no causal relationships have been established.

As part of our findings, we found a negative association between functional capacity and the *Microbacterium* genus. To the best of our knowledge, no microbiome study has established the presence of the *Microbacterium* genus in HF patients. *Microbacterium* is a Gram-positive, non-spore-forming genus within the Actinobacteria phylum that is commonly found in environmental sources such as soil, plants, and fermented foods, although its relevance to heart failure remains largely unclear [46,47,48]. Several *Microbacterium* strains have been shown to improve soil quality by reducing heavy metal concentrations. For example, plant studies have reported higher iron levels alongside reductions in zinc, cadmium, and lead following inoculation with certain *Microbacterium* isolates [47]. These bacteria have also been associated with enhanced drought tolerance and improved plant growth in environments burdened by heavy metal contamination [48].

Some human studies have linked *Microbacterium* to several clinical contexts, including cases of peritonitis in individuals undergoing peritoneal dialysis, in oral cavities, and catheter-associated bloodstream infections [49,50,51]. *Microbacterium* has also been reported in individuals with cystic fibrosis [52], sickle cell [53], and leukemia [54]. Furthermore, *Microbacterium* has also been detected in studies of the cervical microbiome, including among pregnant women who experienced preterm labor [55]. Patterns linking leukemia, sickle cell disease, and preterm labor with disrupted iron levels, together with the high prevalence of iron deficiency in heart failure, raise the possibility that disturbances in iron regulation could be a unifying factor underlying the presence of *Microbacterium* across these conditions. Despite these reports, metagenomic and functional data on *Microbacterium* remain limited, leaving its role in these conditions uncertain. Because our study lacked functional profiling, we could not assess its potential relevance in participants with heart failure. We speculate that patterns linking leukemia, sickle cell disease, and preterm labor with disrupted iron levels [56,57,58], together with the high prevalence of iron deficiency in heart failure [59], raise the possibility that *Microbacterium* may be associated with iron regulation. Further functional analysis is needed to confirm our hypothesis.

Cardiac rehabilitation provides multidimensional benefits that extend beyond traditional measures of systolic function. Emerging evidence from exercise physiology and microbiome science reinforces this broader therapeutic impact. Improvements in myocardial strain parameters, including left and right ventricular global longitudinal strain and left atrial strain, demonstrate that CR can enhance subclinical myocardial performance even when ejection fraction remains unchanged [5,60]. These strain-based indices capture early contractile recovery and ventricular–atrial interaction, offering a more sensitive reflection of the hemodynamic benefits of exercise training than LVEF, which is load-dependent and often insensitive to subtle biological improvements [60,61]. This pattern parallels findings in exercise–gut microbiome research, where functional and biological adaptations occur independently of traditional cardiometabolic markers.

Since the landmark observations by Clarke and colleagues [35], multiple studies have shown that exercise exerts a direct, independent influence on gut microbiota composition. Estaki et al. reported that cardiorespiratory fitness explained more than 20% of the variance in gut microbial diversity, independent of diet, and was associated with enrichment of butyrate-producing taxa such as *Clostridiales*, *Roseburia*, *Lachnospiraceae*, and *Erysipelotrichaceae* [62]. Similar exercise-induced microbial benefits have been documented in older adults, including reductions in potentially pathogenic species such as *C. difficile* and increases in overall microbial diversity following moderate aerobic training [36,37]. Studies consistently show that individuals with HF exhibit lower functional capacity, a gut microbiota characterized by reduced bacterial richness, an overrepresentation of potentially pathogenic taxa, and a depletion of butyrate-producing organisms [6,7,41,63]. Experimental models further support a bidirectional relationship between exercise and the gut–heart axis. In post-myocardial infarction animals, gut microbiota has been shown to mediate the cardioprotective effects of exercise, with exercise training both improving cardiac function and reshaping microbial composition [64,65].

These findings are particularly relevant for individuals with heart failure, who are often older, frail, and unable to engage in vigorous exercise. Yet, even moderate-intensity endurance training, such as that delivered through structured cardiac rehabilitation, has been shown to produce meaningful clinical benefits in this population [66,67]. The convergence of evidence across myocardial mechanics and gut microbial studies suggests that CR may promote both cardiac and extracardiac biological recovery, with improvements in functional capacity, myocardial strain, and microbial composition emerging even in the absence of changes in ejection fraction. This multidimensional response underscores the broader physiological reach of cardiac rehabilitation and highlights the importance of evaluating outcomes beyond traditional systolic metrics.

## 5. Conclusions

Our results indicate that higher functional capacity is associated with differences in gut microbiota composition in HF patients before and after CR. Notably, a clinically meaningful increase in functional capacity was associated with variation in specific microbial taxa, suggesting that HF patients who achieve greater functional capacity may exhibit distinct microbial patterns. These observations point to a potential relationship between functional capacity and gut microbial composition in the context of CR, without establishing directionality or causality. Larger, adequately powered studies are needed to clarify the nature and magnitude of these associations. Taken together, these findings contribute to the growing body of work on the gut–heart axis and offer additional insight into how functional status and microbial ecology may be interconnected in HF.

## 6. Limitations

This study is an exploratory secondary analysis with hypothesis-generating findings. We acknowledge that this study had some limitations that should be considered when interpreting the results. First, dietary intake was not strictly controlled. Although no significant changes in self-reported dietary intake were observed over the study period, diet remains a major confounder in gut microbiota research, and reliance on self-reported dietary measures introduces the potential for underestimation of consumption [68].

Methodologically, gut microbiota assessment relied on 16S rRNA sequencing, which has inherent limitations, including sensitivity to upstream processing variability, limited taxonomic resolution at the genus and species levels, and inability to characterize microbial functional or metabolic profiles [69,70]. Consequently, not all microbial taxa may have been captured.

Finally, the correlational design and use of secondary data precluded assessment of causal relationships between cardiac rehabilitation (CR) and changes in gut microbiota, and the absence of a well-defined control group limited attribution of observed differences to CR exposure. In addition, the non-randomized design and relatively small sample size may have reduced statistical power to adequately detect associations between microbiota composition and functional capacity changes. Additionally, the exercise training was individualized and adjusted according to each participant’s tolerance, which likely contributed to variations in training intensity. Although there was no significant change in BMI pre-post CR in our sample, we recognize that participants still experienced a slight decrease in their BMI. As such, changes in BMI could be a confounding factor in our results. Future studies incorporating whole-genome or metagenomic sequencing with appropriate control groups are needed to clarify how cardiac rehabilitation relates to shifts in gut microbiota and their functional roles.

## Figures and Tables

**Table 1:** Descriptive statistics for sociodemographic and changes in clinical variables for study sample.

Characteristic	Baseline (Pre-CR) M or %	Baseline SD	3-Months (Post-CR) M or %	Post SD	*p* (Paired *t*-Test)
Age (years)	68.0	10.2	68.0	10.2	–
Male (%)	63.4	–	63.4	–	–
Female (%)	36.6	–	36.6	–	–
Caucasian (%)	75.6	–	75.6	–	–
Black (%)	22.0	–	22.0	–	–
Asian (%)	2.4	–	2.4	–	–
NYHA I (%)	29.3	–	29.3	–	–
NYHA II (%)	56.1	–	56.1	–	–
NYHA III (%)	14.6	–	14.6	–	–
Hypertension (%)	100	–	100	–	–
Hyperlipidemia (%)	95.1	–	95.1	–	–
Diabetes (%)	53.6	–	53.6	–	–
Ejection fraction (%)	45.2	17.0	45.2	17.0	–
BMI (kg/m^2^)	32.6	6.7	30.1	10.9	0.57
HDL (mg/dL)	46.7	14.7	52.5	16.6	0.08
LDL (mg/dL)	80.2	35.8	61.9	30.5	0.006
Cholesterol (mg/dL)	155.6	42.7	145.8	42.7	0.001
Triglycerides (mg/dL)	166.1	140.9	164.5	86.5	0.85
Urine sodium (mEq/L)	66.8	33.7	68.7	41.3	0.57
Interleukin-10	4.0	1.4	3.9	1.5	0.56
Interleukin-B	0.2	0.1	0.16	0.08	0.06
Interleukin-6	4.7	3.3	5.1	5.2	0.75
TNF-α	10.1	8.0	9.9	7.1	0.46

**Table 2:** Gut microbiota compositional changes for cohort before and after CR at the phylum level.

Phylum	Pre-CR OTU Abundances	Post-CR OTU Abundances	Paired *t*-Test	
			Statistic	*p* Value
Firmicutes	2,878,676	2,388,199	−04.27	0.0001
Bacteroidetes	2,034,578	1,956,241	−0.56	0.057
Proteobacteria	333,791	822,514	3.04	0.004
Actinobacteria	50,919	109,729	1.64	0.11
F/B ratio	1.41	1.22	−0.38	0.71

Note. OTU = Operational Taxonomic Units; CR = cardiac rehabilitation.

## Data Availability

The data underlying this study cannot be shared publicly because of privacy and ethical constraints, and the original Institutional Review Board protocol did not permit open data release. Access may be granted upon reasonable request to the corresponding author, pending approval from the participating institution and the Institutional Review Board.

## Abbreviations

The following abbreviations are used in this manuscript:

HF	Heart failure
CR	Cardiac rehabilitation
6MWD	Six-minute walk distance
6MWT	Six-minute walk test
CAH	Critical access hospital
SCFA	Short-chain fatty acid
QoL	Quality of life
NYHA	New York Heart Association
BMI	Body Mass Index
HDL	High-density lipoprotein
LDL	Low-density lipoprotein
OTUs	Operational Taxonomic Units
PERMANOVA	Permutational Multivariate Analysis of Variance
PERDISP	Permutational Multivariate Analysis of Dispersion
ANOSIM	Analysis of similarities
PCoA	Principal coordinates analysis
LEfSe	Linear discriminant effect size
FDR	False Discovery Rate
RLE	Relative log expression
PCR	Polymerase chain reaction
DNA	Deoxyribonucleic acid
RNA	Ribonucleic acid
CTAB/SDS	Cetyltrimethylammonium bromide/Sodium dodecyl sulfate
